# Reference values of fractional excretion of exhaled nitric oxide among non-smokers and current smokers

**DOI:** 10.1186/s12890-017-0456-9

**Published:** 2017-08-25

**Authors:** Kjell Torén, Nicola Murgia, Linus Schiöler, Björn Bake, Anna-Carin Olin

**Affiliations:** 1 Section of Occupational and Environmental Medicine, Institute of Medicine, Sahlgrenska Academy, University of Gothenburg, Gothenburg, Sweden; 2 Section of Occupational medicine, Respiratory Diseases and Toxicology University of Perugia, Perugia, Italy; 3 Department of Respiratory Medicine and Allergology, Sahlgrenska University Hospital, Gothenburg, Sweden

**Keywords:** Asthma, Atopy, FE_NO_, General population, Nitric oxide, Normal values, Epidemiology

## Abstract

**Background:**

Fractional exhaled nitric oxide (FE_NO_) is used to assess of airway inflammation; diagnose asthma and monitor adherence to advised therapy. Reliable and accurate reference values for FE_NO_ are needed for both non-smoking and current smoking adults in the clinical setting. The present study was performed to establish reference adult FE_NO_ values among never-smokers, former smokers and current smokers.

**Methods:**

FE_NO_ was measured in 5265 subjects aged 25–75 years in a general-population study, using a chemiluminescence (Niox ™) analyser according to the guidelines of the American Thoracic Society and the European Respiratory Society. Atopy was based on the presence of immunoglobulin E (IgE) antibodies to common inhalant allergens (measured using Phadiatop® test). Spirometry without bronchodilation was performed and forced vital capacity (FVC), forced expired volume in 1 s (FEV_1_) and the ratio of FEV_1_ to FVC values were obtained. After excluding subjects with asthma, chronic bronchitis, spirometric airway obstruction and current cold, 3378 subjects remained. Equations for predictions of FE_NO_ values were modelled using nonparametric regression models.

**Results:**

FE_NO_ levels were similar in never-smokers and former smokers, and these two groups were therefore merged into a group termed “non-smokers”. Reference equations, including the 5th and 95th percentiles, were generated for female and male non-smokers, based on age, height and atopy. Regression models for current smokers were unstable. Hence, the proposed reference values for current smokers are based on the univariate distribution of FE_NO_ and fixed cut-off limits.

**Conclusions:**

Reference values for FE_NO_ among respiratory healthy non-smokers should be outlined stratified for gender using individual reference values. For current smokers separate cut-off limits are proposed.

**Electronic supplementary material:**

The online version of this article (doi:10.1186/s12890-017-0456-9) contains supplementary material, which is available to authorized users.

## Background

In 2005, the European Respiratory Society (ERS) and the American Thoracic Society (ATS) jointly published recommendations for measuring the fractional excretion of exhaled nitric oxide (FE_NO_). These recommendations were followed in 2011 by an updated ATS document on the clinical use of FE_NO_ [1, 2]. The 2011 guidelines suggested that an FE_NO_ level of >50 ppb indicated a high probability of eosinophilic airways inflammation, and that an FE_NO_ level 25–50 ppb should be evaluated further [2]. Subsequent publications have favoured the use of fixed cut-off limits to diagnose asthma, especially Th-2 driven airway inflammation, the most common motive for measuring FE_NO_ [3–5]. To date, however, no consensus has been reached regarding the reference values for FE_NO_, although there is a tendency to support the use of fixed cut off limits [1, 6].

Moreover, there is a lack of knowledge regrading reference values in current smokers. A study published in 1995 reported that smokers exhaled lower concentrations of nitric oxide (NO) than non-smokers [7]. This finding has since been confirmed in other studies [8–10]. The usefulness of FE_NO_ values in assessing asthma and Th-2 driven airway inflammation in smokers is still unclear, although some studies have shown that smokers with asthma had higher FE_NO_ levels than healthy subjects who smoked [11]. Hence, there is a need to determine reference values for FE_NO_ among current smokers.

The primary aim of the present study was to establish reference values for FE_NO_ at an exhalation flow rate of 50 mL/s based on a large random population comprising never-smokers, former smokers and current smokers. The second aim of the study was to determine whether age, sex, height and atopy should to be considered when proposing reference values. The analysis was based on an expanded population sample of 5265 subjects; one segment of the cohort, 1803 never-smokers, was included in our previous paper [12].

## Methods

The data for this study were extracted from the previously described ADONIX (Adult-Onset Asthma and Exhaled Nitric Oxide) random general-population study in Sweden, which included individuals aged 25–75 years [8, 12]. The clinical measurements were performed between 2002 and 2007. We included subjects with complete anthropometric data, smoking data, spirometry data and FE_NO_ levels measured at an exhalation flow rate of 50 mL/s (*n* = 5854). The FE_NO_ measurements were performed before spirometry. After excluding subjects whose FE_NO_ measurements did not meet the quality criteria (see below) 5265 subjects remained in the cohort.

FE_NO_ was measured with a chemiluminescence analyzer (NIOX, Aerocrine AB, Stockholm, Sweden) and the analyser was calibrated every other week with a certified calibration gas. All procedures were performed in accordance with the recent ATS recommendations and have been described previously in detail [13, 14]. Briefly, the subjects exhaled against a mouth pressure of 5 cm H_2_O at 50 mL/s for 10 s. NO was measured between the 6th and 10th second. Subjects were excluded if the exhaled flow was >55 mL/s or <45 mL/s. Blood samples were analysed for the presence of immunoglobulin E (IgE) antibodies to common inhalant allergens (Phadiatop, Pharmacia, Uppsala, Sweden) and the results were classified as negative (class 0) or positive (class 1) [15].

Height and weight were measured with light clothing and without shoes. Spirometry without bronchodilation was performed after the FE_NO_ measurements using a dry-wedge spirometer (Vitalograph, Maids Moreton, UK) and according to the ATS/ERS standards [16]. The forced vital capacity (FVC) and the forced expired volume in 1 s (FEV_1_) were obtained with the subjects in a sitting position, wearing a nose clip. The ratio of FEV_1_ to forced vital capacity (FVC) was calculated and expressed as FEV_1_/FVC%). Predicted normal values for the spirometric variables were obtained from the same population [17].

The subjects were classified as never-smokers, former smokers and current smokers, based on their responses to a questionnaire, as previously described [8]. A smoker who had refrained from smoking for >1 year was considered as a former smoker. Affirmative responses to the following questions or positive test results were used to define asthma, chronic bronchitis, atopy, and cold;

Asthma; Do you have or have you ever had asthma? *or* Have you ever had asthma diagnosed by a physician? [18].

Chronic bronchitis; Have you had persistent cough since the age of 15? *and* If yes, did any coughing period last at least 3 months? *and* If yes, have you experienced such coughing periods for at least two consecutive years? [19].

Atopy; Presence of IgE antibodies in blood sample tested using Phadiatop (class 1) [15].

Cold; Do you have a cold now? *and/or* Do you have a sore throat now?

Airway obstruction; FEV_1_/FVC ratio below the lower limit of normal [17].

We excluded subjects with asthma, chronic bronchitis, airway obstruction, and a cold, 3378 subjects remained in the cohort.

Statistical analyses were performed using SAS (Statistical Analysis System, version 9.3; SAS Institute Inc.; NC, USA). All comparisons between groups were analysed by the Mann-Whitney U test and two-sided *p*-values are presented. FE_NO_ levels were not normally distributed; hence all univariate analyses were performed using nonparametric tests. In preliminary multiple regression analyses on ln FE_NO_ the explanatory variables age, height, weight, body mass index (BMI) and atopy were included. Age, height and atopy were significant for both men and women and were therefore kept in the final multiple regression models (QUANTREG procedure) to obtain the estimated coefficients for median, 5th and 95th percentiles. To test whether the effect of age and height on FE_NO_ differed between never-smokers and former smokers we included smoking status, height, age, and the interactions between smoking status and both age and height. There was no significant effect of smoking status.

## Results

Descriptive data, including FE_NO_ values for 3378 respiratory healthy subjects, are presented in Table 1. Median FE_NO_ values and the 5th and 95th percentiles according to smoking status and sex are presented in Table 2. In the whole population the median FE_NO_ value was 16.5 ppb with 7.2 ppb and 39.0 ppb as the 5th and 95th percentiles, respectively. FE_NO_ levels were significantly (*p* < 0.0001) higher in men than in women. Further, current smokers had significantly (*p* < 0.05) lower FE_NO_ levels (by around 6 ppb) than former smokers or never-smokers. FE_NO_ levels were similar among never-smokers and former smokers. In addition, linear regression did not find a significant effect of smoking status (never-smoker/former smoker) among women or men (data not shown). Hence, never-smokers and former smokers were merged into a group termed as “non-smokers” in subsequent analyses.

**Table 1:** General characteristics and lung function of 3378 randomly selected healthy subjects

	All (*n* = 3378)		Women (*n* = 1741)		Men (*n* = 1637)	
	Mean	SD	Mean	SD	Mean	SD
Age (years)	51.4	11.2	50.9	11.3	52.0	11.1
Height (cm)	172.4	9.3	166.2	6.5	179.0	6.9
Weight (kg)	77.6	14.4	70.2	12.0	85.5	12.4
BMI (kg/m_2_)	26.0	3.9	25.4	4.2	26.7	3.5
FVC (% pred)	98.1	11.9	99.1	11.8	97.0	11.9
FEV_1_ (% pred)	98.6	12.1	99.7	12.0	97.6	12.1
FEV_1_/FVC (%)	79.5	4.7	79.7	4.7	79.3	4.7
FEV_1_/FVC (% pred)	100.5	5.6	100.4	5.4	100.6	5.7

Mean values and standard deviations (SD) are presented

**Table 2:** FE_NO_ (ppb) of respiratory healthy subjects (*n* = 3378) according to smoking habits

Smoking groups	Women (*n* = 1741)				Men (*n* = 1637)				
	*n*	Median	5th perc.	95th perc.	*n*	Median	5th perc.	95th perc.	*p* value^a^
Never smokers	868	15.7	7.8	35.7	817	19.0	9.0	44.2	<0.0001
Former smokers	581	16.3	7.6	35.6	615	18.9	9.2	39.9	<0.0001
Current smokers	292	10.4^b^	4.4	29.4	205	13.2 ^b^	6.2	34.3	<0.0001
All subjects	1741	15.0	6.6	35.3	1637	18.2	8.2	41.3	<0.001.

^a^ *p* values refer to differences between median values of FE_NO_ according to the Mann-Whitney

two sided test

^b^denotes a significant difference from the other smoking categories ANOVA)

Number of subjects, median values and the 5th and 95th percentiles of FE_NO_ (ppb) are presented

Table 3 shows the FE_NO_ values for men and women stratified by age groups. Irrespective of age group, FE_NO_ levels were significantly (*p* < 0.05) higher among men than women. Furthermore, FE_NO_ levels increased with age among both men and women.

**Table 3:** FE_NO_ results (ppb) of non-smokers, i e never-smokers and former smokers united (*n* = 2881), of the ADONIX database subdivided according to age class and sex

Age class (years)	Women (*n* = 1449)				Men (*n* = 1432)				*p*
	*n*	Median	5th perc	95th perc	*n*	Median	5th perc	95th perc	
25–34	103	12.9	5.9	30.3	88	16.8	9.4	39.5	<.0001
35–44	320	14.3	6.8	31.3	296	17.5	7.9	38.9	<.0001
45–54	406	15.4	7.7	33.6	417	19.1	8.8	41.7	<.0001
55–64	411	18.1	8.3	37.9	418	20.0	9.3	43.2	0.010
>64	209	19.5	8.3	42.2	213	21.1	9.2	50.6	0.028

*p* values refer to differences between median values according to the Mann-Whitney two- sided test

Number of subjects, median values and the 5th and 95th percentiles are presented

FE_NO_ values for non-smokers stratified by atopy and sex are presented in Table 4. FE_NO_ levels were significantly (*p* < 0.05) higher (by around 2 ppb) among subjects with atopy.

**Table 4:** FE_NO_ of healthy subjects of the ADONIX database (*n* = 3378) subdivided into subsets according to the outcome of the Phadiatop test

Subsets	Phadiatop negative				Phadiatop positive				*p*
	*N*	Median	5th perc	95th perc	*N*	Median	5th perc	95th perc	
All	2210	16.9	7.9	36.7	671	19.1	8.8	49.7	<.0001
Women	1133	15.7	7.7	33.6	316	17.1	7.5	46.1	0.0005
Men	1077	18.4	9.0	39.2	355	20.7	9.8	52.7	<.0001

*p* values refer to differences between median values of FE_NO_ according to the Mann-Whitney two sided test

# denotes a significant difference from the other smoking categories (ANOVA)

Number of subjects, median values and the 5th and 95th percentiles of FE_NO_ (ppb) are presented

**Fig. 1:**
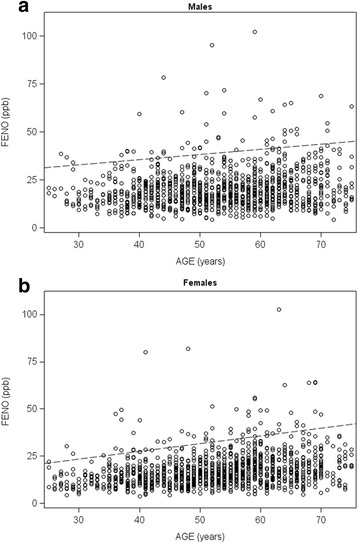
Plots of FE_NO_ against age for men (Fig. 1a) and women (Fig. 1b), respectively, which illustrate the age dependency of the upper limit normal. Only subjects with negative Phadiatop® results are presented (*n* = 2210). The y-axis range is restricted and excludes one female subject and three male subjects with FE_NO_ above 110 ppm. The *dashed lines* indicate the upper 95th percentiles assuming height of women and men to be 166 and 179 cm respectively

Table 5 presents FE_NO_ reference equations for men and women including median values and the 5th and 95th percentiles from quantile regression models for non-smokers (never-smokers and former smokers). Individual FE_NO_ values for men and women are plotted in Fig. 1a and b. The dashed lines represent the 95th percentile and the age dependence of the upper limit of normal, particularly among women. As an example, the predicted 95th percentile of FE_NO_ values for a 40 year-old non-atopic woman, height 170 cm, is 4.0 + 0.4081*40 + 0.1414*170–16.1097 = 28.2 ppb.

**Table 5:** Coefficient estimates of reference equations for FE_NO_. The 5th percentile, median value and the 95th percentiles are presented for females and males respectively

	Women (*n* = 1406)			Men (*n* = 1410)		
	5th perc.	Median	95th perc.	5th perc.	Median	95th perc.
Intercept	−6.9	−12.7	4.0	−5.1	−14.1	84.7
Age (years)	0.0629	0.2205	0.4081	0.0508	0.1444	0.2682
Height (cm)	0.0736	0.1189	0.1414	0.0694	0.1559	−0.2501
Phadiatop positive	0.0	0.0	0.0	0.0	0.0	0.0
Phadiatop negative	−0.9	−2.7	−16.1	−1.1	−2.8	−15.0

Regression modelling for the 420 current smokers in the cohort resulted in unstable models. Hence, the proposed reference values for current smokers are based on the univariate distribution of FE_NO_ values and 95th and 5th percentiles shown in Table 2. The proposed upper reference value for FE_NO_ is 29.4 ppb for female current smokers and 34.3 ppb for male current smokers.

The Additional file 1 includes an Excel-based FE_NO_ calculator for obtaining individual predicted reference values (median and 5th and 95th percentiles) after entering sex, age and height; Phadiatop blood test results (positive or negative) are an optional variable.

## Discussion

The present study found that FE_NO_ levels among non-smokers are significantly influenced by sex, height, age and atopy, and the reference equations imply that considerable differences in the reference values between a young woman and an older man would be considerable. The reference equations give the upper reference value for a non-atopic woman, 30 years old and 160 cm tall, as 23 ppb, and for a non-atopic man, 60 years old and 180 cm tall, as 41 ppb. Hence, we consider that the findings support the use of individual reference values among non-smokers rather than fixed cut-off limits. However, among current smokers fixed cut-off limits should still be used.

One approach to establishing criteria of normality has been the use of fixed cut-off values for abnormality based on the distribution among normal healthy individuals. Another approach is the production of reference values based on regression equations obtained from the distribution of factors of interest and important predictors, often anthropometric factors. However, the explanatory value of anthropometric factors for FE_NO_ is rather low, and that is one reason why cut-off values have often been recommended. The present results indicate, however, that there are considerable differences in the predicted reference values, which support the use of individual reference values based reference equations instead of a fixed cut-off value.

Whether subjects with atopy should be included in a healthy reference population is not clear. Most published studies have included subjects with atopy [2, 12, 20, 21]. At present, the clinical significance of increased production of specific IgE antibodies in non-asthmatic subjects is unclear, but certainly does not imply the presence of disease. Phadiatop test has been shown to exhibit higher sensitivity but lower specificity than the skin-prick test identifying subjects with allergic manifestations such as asthma, dermatitis or rhinitis [15]. However, the clinical significance is unclear. Subjects with atopy (positive Phadiatop test) had a 2 ppb higher median FE_NO_, 13 ppb higher 95th percentile, Table 4. We therefore consider it important to present reference equations separately for atopics and non-atopics, which in the present study was based on Phadiatop testing. In a clinical situation with no information on the patient’s atopic status, we recommend to applying the reference equation for non-atopics (negative Phadiatop). We stress that a positive Phadiatop is not a determination of atopic disease. We should also add that our study population comprised subjects with atopic dermatitis, a condition that has been associated with increased FE_NO_ levels [22].

From the present results we can conclude that there are consistent differences between men and women, as women had lower FE_NO_ levels than men. The sex difference was not observed in the 1131 subjects included in our previous paper [12]. However, a sex difference was also numerically present in the previous study, but did not reach statistical significance because of the smaller sample-size. The lower FE_NO_ levels observed in women may not necessarily reflect lower NO production in women, as FE_NO_ is highly flow-dependent, and decreases substantially with increasing flow. For any given exhalation-flow from the mouth (we used 50 mL/s), the linear flow velocities within the airways will depend on lung size, and the smaller the lung the higher the linear flow velocities. As women have smaller lungs and consequently higher linear flow velocities in the airways, lower FE_NO_ levels may be expected. Furthermore, the airway surface area that produces NO is smaller among women. Accordingly, the sex-observed for FE_NO_ values in healthy subjects may to some extent be attributable to airway size difference rather than to differences in NO production.

The proposed reference values reflect the distribution of FE_NO_ levels, and may serve as a tool to enhance the identification of subjects with deviating, abnormal values, and possibly on-going Th-2 driven inflammation. The usefulness of reference values for FE_NO_ is indeed to determine what is abnormal. When using FE_NO_ to diagnose asthma, clinicians may also consider other factors. For a correct diagnosis, FE_NO_ values with the highest positive- and negative likelihood ratios must be identified, and those may be different for Th-2 and non-Th-2 driven asthma. However, the present study was not designed to define these diagnostic characteristics, and we lack other markers of T-helper-2 driven inflammation, for example in induced-sputum, for comparison. The accuracy of different fixed cut-off limits in relation to clinical asthma has recently been evaluated, showing that increasing cut-off limits increase the specificity [6]. These authors concluded that a reasonable balance between specificity and sensitivity would be a cut-off value >45 ppb. For non-smokers, we suggest the use of the individual upper 95th and lower 5th percentiles based on sex, age, atopy and height. The upper limits of normal calculated from the present findings are substantially lower than the upper limits suggested for diagnosing eosinophilic airways inflammation, suggested by the American Thoracic Society [2]. The ATS suggests that an FE_NO_ value exceeding 50 ppb indicates a high probability of eosinophilic airway-inflammation, and values of 25–50 ppb require further evaluation. However, our findings indicate that the upper limits of what is apparently normal can differ greatly between a young woman without atopy (around 22 ppb) and an elderly man (over 40 ppb). When evaluating FE_NO_ in clinical practice the presented upper limits of normal are important.

We consider the present proposed reference values for current smokers to be original. We confirmed results from previous studies, which reported that current smokers exhibited low FE_NO_ levels [23]. Several potential mechanisms underlying this finding have been proposed, such as the hypotheses that smoking may induce lower FE_NO_ metabolism or that smoking may induce a reduced active in nitric oxide synthases in the airways [23]. We modelled the data with various anthropometric variables, but because of the unstable models, we decided to propose sex-specific fixed cut-off limits based on a univariate analysis of our data. Further studies are needed to evaluate whether FE_NO_ levels in current smokers have clinical relevance [11].

Low FE_NO_ levels are of increasing interest. A study published in 1997 showed that 27 patients with cystic fibrosis had low FE_NO_ levels, with a mean level of 5.9 ppb [24]. FE_NO_ levels have also been studied in relation to BMI; low levels were observed in both obese and underweight individuals [25, 26]. Moreover, low FE_NO_ levels have been discussed in relation to certain asthma phenotypes [27]. These reports underscore the need to establish reference limits that define low levels of FE_NO_. The present reference equations provide the lower limits of normal and ought to be of help in identifying subjects with abnormally low FE_NO_.

Several small general-population studies from different countries have calculated reference values for FE_NO_ [4, 10, 12, 20, 21, 28–30]. There is, however, one large recently published general-population study from the US (the National Health and Nutrition Examination Survey 2007–2010) where the recommended cut-off values for diagnosing asthma in individuals aged 12–80 years was 39 ppb based on the 95th percentile, i.e. the upper and lower 2.5th percentiles [3]. The authors recommended the same value for men and women, and the statistical analyses were designed quite differently from the present study. The US study excluded only subjects with asthma and wheezing, but included smokers and subjects with atopy. The final regression model included age, height, sex, race, smoking status and passive smoking.

The present study was performed in a random adult population with a participation of approximately 40%; the highest non-participation was observed in young men and in smokers [31]. A possible selection bias may have been present and affected our analysis, as current smokers were overrepresented among non-participants. Another issue to note is that certain conditions which may affect the levels of FE_NO_, such as cystic fibrosis and atopic dermatitis, were not excluded [22, 24]. Furthermore, we did not exclude subjects using inhaled or oral corticosteroids. However, this was a random population sample and subjects with asthma, airway obstruction and chronic bronchitis were excluded, reducing the bias in relation to inhaled or oral corticosteroids.

## Conclusions

We conclude that the present reference values for female and male non-smokers (never-smokers and former smokers) that are based on age, height and atopy are more appropriate than fixed cut-off limits. However, for current smokers separate cut-off limits should still be used.

## Additional file

Additional file 1: Excel-based FE_NO_ calculator. (XLSX 19 kb)

## Abbreviations

ADONIX: Adult-onset asthma and exhaled nitric oxide
ATS: American thoracic society
BMI: Body mass index
ERS: European respiratory society
FE_NO_: Fractional excretion of exhaled nitric oxide
FEV_1_: Forced expiratory volume in 1 s
FVC: Forced vital capacity
IgE: Immunoglobulin E
NO: Nitric oxide
ppb: Parts per billion
SAS: Statistical analysis system
